# Pocket-sized, wireless-Bluetooth ultrasound system to perform diagnostic and low-complexity interventional procedures in bedridden patients during the COVID-19 pandemic: from intensive care unit to domiciliary service?

**DOI:** 10.1186/s41747-022-00273-1

**Published:** 2022-05-10

**Authors:** Christian Ossola, Filippo Piacentino, Federico Fontana, Marco Curti, Giada Zorzetto, Andrea Coppola, Giulio Carcano, Massimo Venturini

**Affiliations:** 1 Department of Diagnostic and Interventional Radiology, Circolo Hospital and Macchi Foundation, Insubria University, Varese, Italy; 2 Department of General, Emergency and Transplants Surgery, Circolo Hospital and Macchi Foundation, Insubria University, Varese, Italy

**Keywords:** Bedridden persons, COVID-19, Mobile applications, Ultrasonography, Wireless technology

## Abstract

The use of a pocked-sized, wireless-Bluetooth ultrasound portable system with display images presented on a tablet facilitated the work of our radiologists during the first wave of coronavirus disease 2019 (COVID-19) to perform diagnostic and interventional procedures in bedridden patients. The device is equipped with a battery-powered probe without cables that transmits images to a tablet (or a cell phone) through a dedicated App. We hypothesise in future to extend diagnostic and low-complexity interventional procedures from hospitalised patients to at-home patients who are not able to mobilise out of bed or are difficult to transport. This domiciliary service might also reduce the overhead of hospital accesses.

## Key points

- Pocket-sized, wireless-Bluetooth ultrasound systems can allow bedside diagnostic and low-complexity interventional procedures.
- Those bedside diagnostic and interventional procedures could be extended from hospitalised to at-home patients.
- This innovative domiciliary service might also reduce the overhead of hospital accesses.

The increase in life expectancy over 80 years in industrialised countries has led to a higher and higher prevalence of chronic diseases that often coexist in the same individual, making its management difficult, leading to not always justified crowding of emergency rooms [1].

This evidence has become more acute during the severe acute respiratory syndrome coronavirus 2 (SARS-CoV-2) pandemic, when the risk of clogging of emergency rooms has been added to the risk of contagion for patients and health workers [2]. The introduction of systems based on wireless, Bluetooth, and touchscreen technologies has recently also included ultrasound imaging [3]. Ultrasound has low cost, is radiation free, operator-dependent, easily repeatable, capable of real-time imaging, allowing rapid diagnosis for example in case of emerging diseases [3, 4]. Ultrasound is also widely used as the main guidance system for various interventional procedures such as biopsies, drainage placements, and ablations [1].

An innovative pocked-sized, wireless-Bluetooth ultrasound system (Clarius Mobile Health Corp, Vancouver, Canada) facilitated the work of radiologists during the first wave of coronavirus disease 2019 (COVID-19) to perform diagnostic and interventional procedures in bedridden patients. The device is equipped with a battery-powered probe without cables and can transmit images to a tablet or cell phone through a dedicated app [2, 5].

**Fig. 1:**
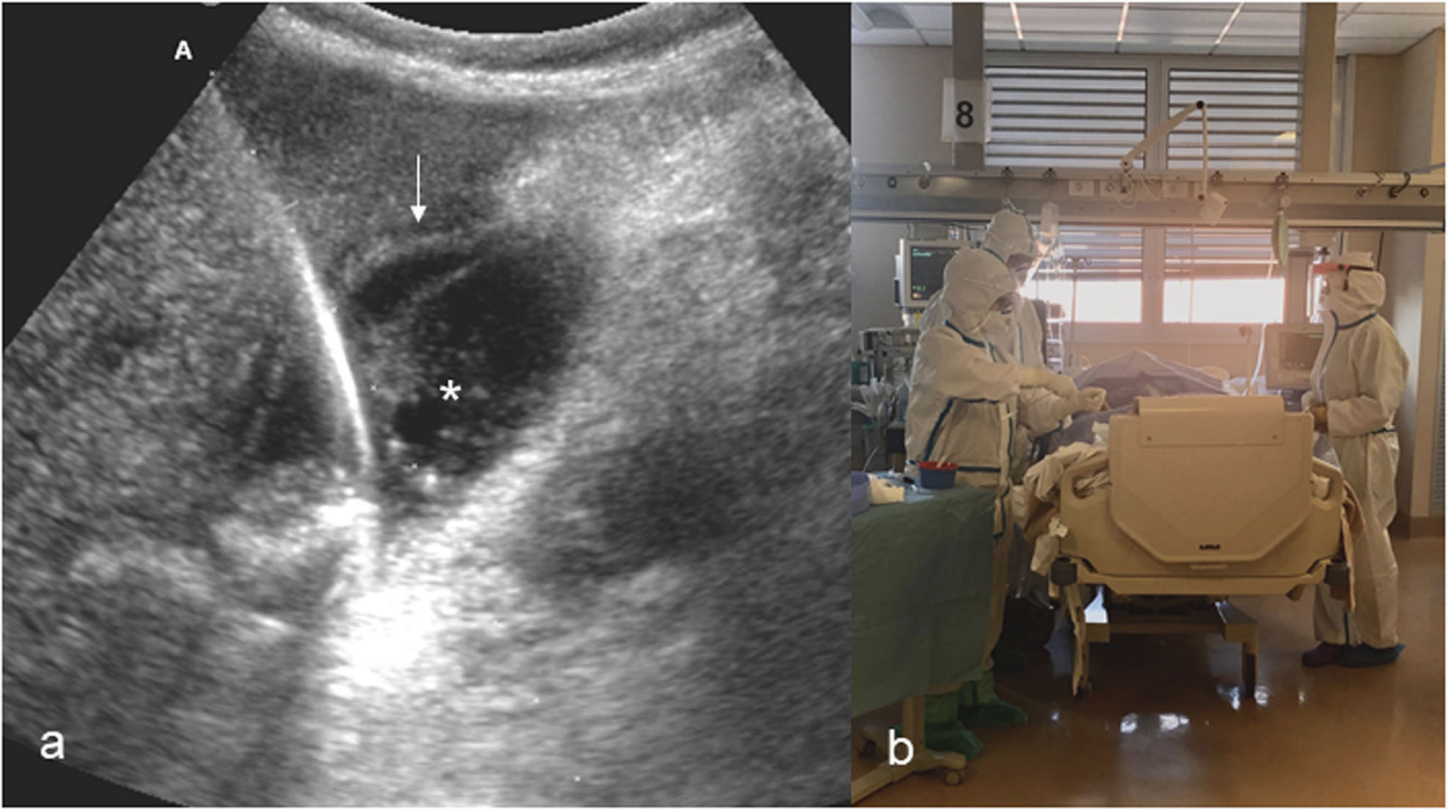
Percutaneous transhepatic ultrasound-guided cholecystostomy through an intercostal approach in a coronavirus disease 2019, COVID-19, patient with acute cholecystitis. The guidewire (A), the “railway sign” of the gallbladder wall (arrow), and the presence of sludge and microlithiasis (asterisk) are shown in **a**. The interventional radiology team performing percutaneous gallbladder drainage at the bedside in intensive care unit is shown in **b**

**Fig. 2:**
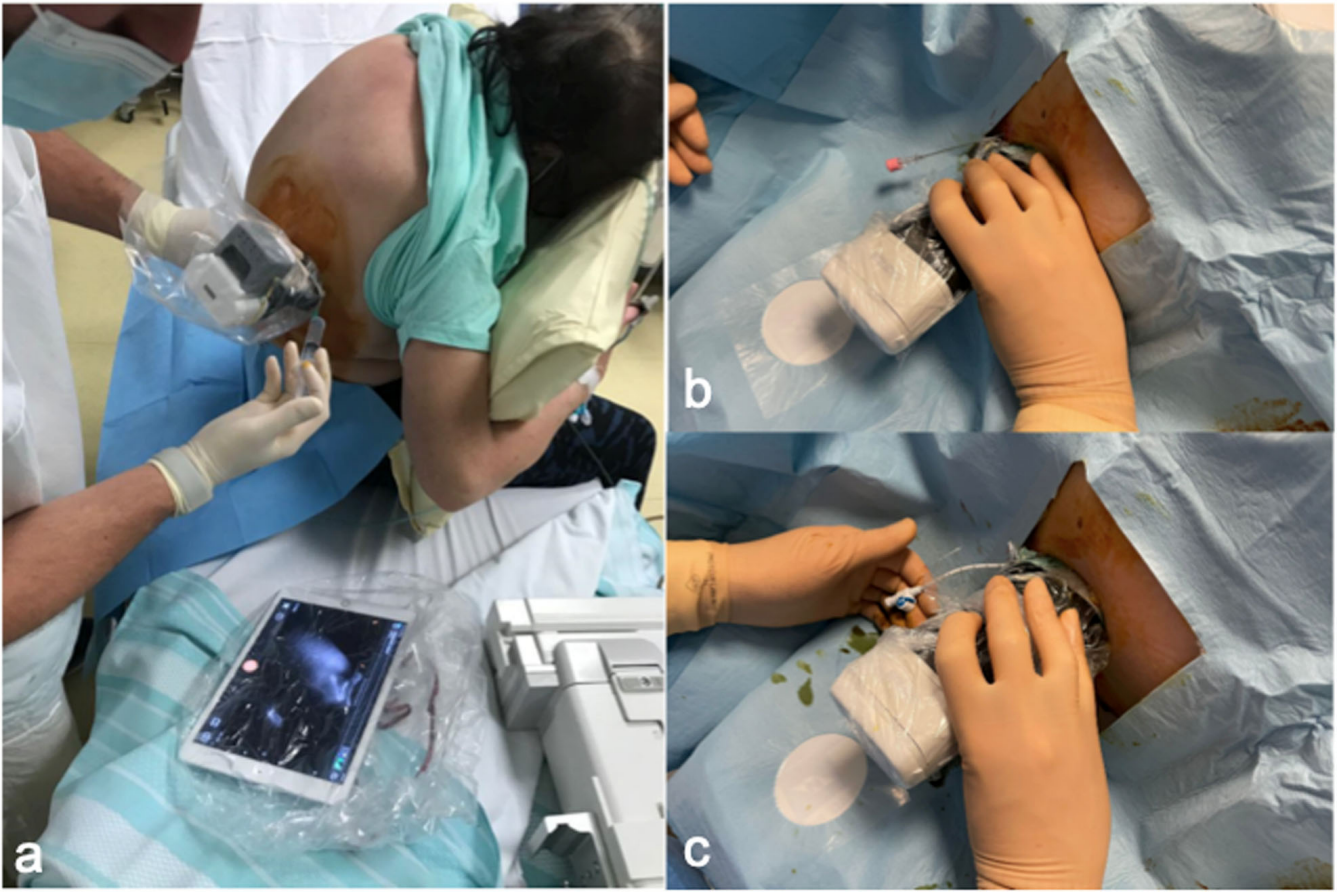
A low-complexity interventional radiology procedure performed at the patient's bedside using a pocket-sized, wireless-Bluetooth ultrasound system. Images show the diagnostic ultrasound of the right pleural cavity displayed on the tablet (**a**) and the placement of the pleural drainage (**b**, **c**)

In our hospital, many patients affected by SARS-CoV-2 developed biliary sludge and subsequently acute cholecystitis. In this inauspicious context, the placement of a percutaneous cholecystostomy in the angiographic room—as usually performed—added three additional issues to an already strained hospital management framework [6]. The first was the transport of patients throughout the hospital, increasing the risk of infection spread. The second was the occupation of an angiographic room which then must be sterilised. The third was the necessity to provide personal protection equipment for the whole team involved in the procedure [1]. To solve these problems, we thought to perform bedside cholecystostomy in COVID-19 patients in the intensive care unit. Transhepatic gallbladder drainage was performed by two interventional radiologists with appropriate personal protection equipment for standard, contact, and airway precautions (Fig. 1) according to the World Health Organization recommendations [7]. Several diagnostic ultrasound examinations and ultrasound-guided interventional procedures such as pleural or peritoneal drainage (Fig. 2) were performed in bedridden patients in the intensive care unit [8]. Typically, pre-pandemic bedside procedures were performed in patients admitted to intensive care units or in patients with medical issues that did not allow their transfer to the interventional radiology room [8].

All these cases were conducted using the pocket-sized, wireless-Bluetooth ultrasound system, with appropriate sterile probe cover and protection system of the cooling fan. The tablet on which the images have been transmitted was also adequately protected. Images were transferred to the local Picture Archiving and Communicating System, permitting the full legal archiving of the images and the possibility of making comparisons over time during proper follow-up [9].

We hypothesise a future scenario where diagnostic and low-complexity interventional procedures (thoracentesis, paracentesis, peritoneal, and pleural drainages) based on pocket-sized ultrasound systems not only in hospitalised but also at-home patients, creating a new domiciliary service [1, 2]. Many elderly, fragile, oncologic, and bedridden patients could benefit from this perspective [2]. This diagnostic and interventional homecare setting leads to a reduced risk of infection for compromised patients, less stress for hospitalisation and better follow-up options.

In the case of an interventional procedure, an interventional radiologist and an experienced nurse should be involved, making the whole procedure easily manageable. These procedures are safe, with life-threatening complications (*e.g.*, haemoperitoneum, bowel perforation, accidental puncture of an arterial vessel along the drainage pathway with progressive aspiration of bright red blood) occurring in very rare cases [1, 10]. At any rate, at the end of the procedure, the patient will remain under observation for at least 30 min. In the case of major complications, the interventional team can alert emergency physicians and transfer immediately the patient to the emergency room of the nearest hospital, possibly that where the interventional team is based.

This innovative domiciliary service might also reduce the overhead of hospital accesses particularly of emergency departments, overloaded with COVID-19 patients during the high waves of the recent pandemic [11]. Moreover, the angiographic room would be available to perform more complex interventions, optimising the resources of the hospital and also of the surrounding territory. In the end, the expected advantages are those of minimising the pressure on emergency rooms, guaranteeing diagnostic and therapeutic procedures to patients who can be transported with difficulty and to implement new guidelines that push towards territorial medicine.

## Data Availability

Not applicable

## Abbreviations

COVID-19: Coronavirus disease 2019
SARS-CoV-2: Severe acute respiratory syndrome coronavirus 2
